# Omega-3 fatty acids for treatment of non-alcoholic fatty liver disease: design and rationale of randomized controlled trial

**DOI:** 10.1186/1471-2431-13-85

**Published:** 2013-05-23

**Authors:** Wojciech Janczyk, Piotr Socha, Dariusz Lebensztejn, Aldona Wierzbicka, Artur Mazur, Joanna Neuhoff-Murawska, Pawel Matusik

**Affiliations:** 1 Department Gastroenterology, Hepatology and Eating Disorders, Children’s Memorial Health Institute, Warsaw, Poland; 2 Department of Pediatrics, Gastroenterology and Allergology, Medical University of Bialystok, Bialystok, Poland; 3 Laboratory Diagnostics, Children's Memorial Health Institute, Warsaw, Poland; 4 Medical Faculty, University of Rzeszow, Rzeszow, Poland; 5 Departament Pediatric Endocrinology and Diabetes, Medical University of Silesia, Katowice, Poland

**Keywords:** Non-alcoholic fatty liver disease, Omega-3 fatty acids, Polyunsaturated fatty acids, Randomized controlled trial, Children

## Abstract

**Background:**

Non-alcoholic fatty liver disease (NAFLD) is a liver manifestation of metabolic syndrome since obesity and insulin resistance are the main pathogenic contributors for both conditions. NAFLD carries increased risk of atherosclerosis and cardiovascular diseases. There is an urgent need to find effective and safe therapy for children and adults with NAFLD. Data from research and clinical studies suggest that omega-3 fatty acids may be beneficial in metabolic syndrome-related conditions and can reduce the risk of cardiovascular disease.

**Methods/design:**

We are conducting a randomized, multicenter, double-blind, placebo-controlled trial of treatment with omega-3 fatty acids in children with NAFLD. Patients are randomized to receive either omega-3 fatty acids containing docosahexaenoic acid (DHA) and eicosapentaenoic acid (EPA) or placebo for 24 weeks. The dose of omega-3 (DHA+ EPA) ranges from 450 to 1300 mg daily. Low calorie diet and increased physical activity are advised and monitored using validated questionnaires. The primary outcome of the trial is the number of patients who decreased ALT activity by ≥ 0,3 of upper limit of normal. The main secondary outcomes are improvement in the laboratory liver tests, liver steatosis on ultrasound, markers of insulin resistance and difference in fat/lean body mass composition after 6 months of intervention.

**Discussion:**

Potential efficacy of omega-3 fatty acids in the treatment of NAFLD will provide needed rationale for use of this safe diet supplement together with weight reduction therapy in the growing population of children with NAFLD.

**Trial registration:**

NCT01547910

## Background

Non-alcoholic fatty liver disease (NAFLD) comprises spectrum of liver damage that ranges from simple steatosis through steatohepatitis (NASH), fibrosis to cirrhosis, end-stage liver disease and occasionally hepatocellular carcinoma. Although the natural history of NAFLD remains yet to be defined, it is now clear that NAFLD and especially NASH carries increased risk of atherosclerosis, cardiovascular disease (CVD) and overall mortality when compared to general population [1-8]. Emerging evidence suggests that the main cause of death in NAFLD is CVD what strongly reflects its very close relation to metabolic syndrome (MS) [1-3]. In patients who develop liver steatosis at younger age the complications of the liver disease and metabolic syndrome may appear sooner, therefore they require special concern and early management.

Weight reduction appears to be the front-line therapy in NAFLD. Dietary restrictions are as important as physical activity and changes in lifestyle [9-12]. However compliance is poor in these patients and there is too much inconsistent data from the clinical trials to indicate the optimal rate and extent of weight loss in children.

Pharmacological treatment remains unsuccessful in both children and adults with NAFLD. In the planning phase of this trial we performed a systematic review of literature on pharmacological interventions for NAFLD [13]. Use of probucol, pioglitazone, metformin and carnitine in high doses may be promising but in conclusion none of the pharmacological agents could be recommended for treatment of NAFLD, especially in children. Recent two large clinical trials using vitamin E and pioglitazone in NAFLD showed conflicting results [14,15].

Omega-3 long-chain polyunsaturated fatty acids are safe diet supplements that showed efficacy in the prevention and therapy of cardiovascular diseases, dyslipidemia and metabolic syndrome [16-19]. In a paucity of effective treatment they may constitute safe pharmacological option for children with NAFLD.

Recently Parker et al. published systematic review and meta-analysis of studies of omega-3 LC-PUFA (long-chain polyunsaturated fatty acids) supplementation in adults with NAFLD [20]. Nine studies (5 RCTs), involving 355 individuals were included. Patients were given either omega-3 LC-PUFA or control treatment. Duration of therapy ranged from 8 weeks to 12 months, as well as the dose of LC-PUFA - from 0,25g to 13,7g per day. In conclusion of the review, omega-3 fatty acids were claimed beneficial vs. placebo in reducing liver fat (estimated by liver biopsy, ultrasound or 1H-MRS) (effect size = ˙0.97, 95% CI: ˙0.58 to ˙1.35, p <0.001) and decreasing AST (effect size = ˙0.97, 95% CI: ˙0.13 to ˙1.82, p = 0.02) but not ALT activity. However the current data are not sufficient to set the optimal dose of LC-PUFA in NAFLD.

So far there has been only one randomized controlled trial that assessed efficacy of LC-PUFA in children with NAFLD [21]. Patients were assigned to receive 250 mg or 500 mg of DHA (docosahexaenoic acid) per day or placebo for 6 months. Beneficial results of DHA were expressed in the improvement of level triglycerides, markers of insulin resistance and liver fat assessed by ultrasound. DHA did not show significant effect on ALT activity.

Below we describe design of our multicenter, randomized, double-blind, placebo-controlled trial of omega-3 LC-PUFA supplementation in children with NAFLD.

## Methods

### Study design overview

We are conducting a multi-center, randomized, placebo-controlled, double-blind clinical trial to assess efficacy of treatment with omega-3 LC-PUFA in children with established NAFLD. Eligible patients are randomized to two arms: these who receive either fish oil containing omega-3 LC-PUFA: docosahexaenoic acid (DHA) and eicosapentaenoic acid (EPA) (450 to 1300 mg daily), or placebo (sunflower oil) for 24 weeks.

**Figure 1:**
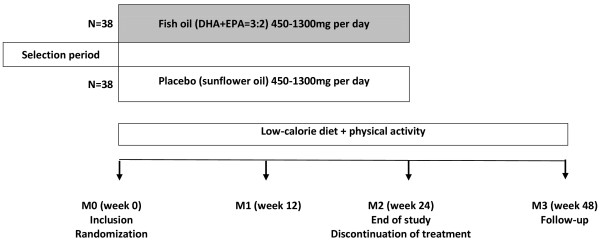
Schematic of the trial design.

The main aim of the study is to determine whether supplementation of omega-3 LC-PUFA improves biochemical parameters of liver function, markers of insulin resistance, serum lipid profile and liver steatosis on ultrasound in patients with non-alcoholic fatty liver disease. Primary comparisons will be made using an intention-to-treat analysis at week 12 and 24. Per protocol analysis will be also applied for the same time points. The follow-up visit is planned after 6 months after discontinuation of study treatment. A schematic of the trial design is shown on Figure 1.

### Participant selection

**Figure 2:**
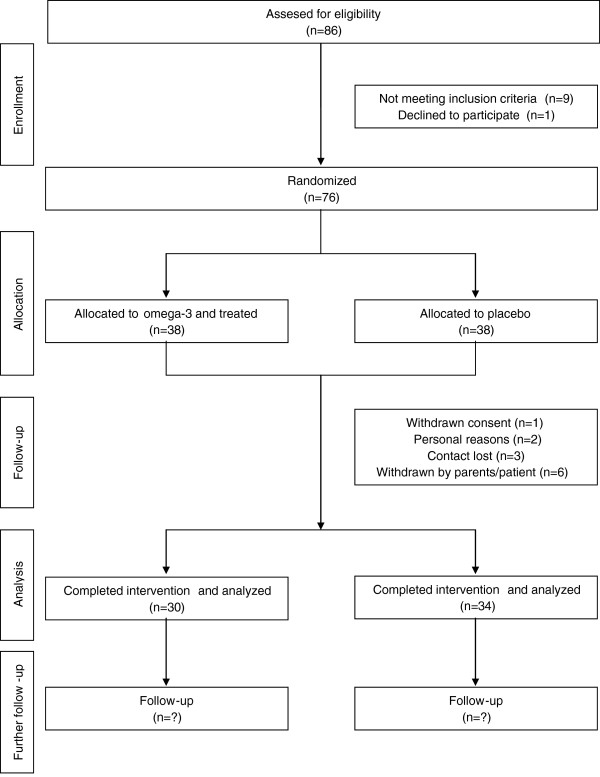
Patient CONSORT flowchart.

Children aged 6 to 19 years old were recruited at the 4 Polish pediatric hospitals from July 2008 till April 2011 (the participating centers are listed in the acknowledgments). In total 76 patients were enrolled into the trial according to the screening procedures. All participants and their parents gave written consent before enrollment. Ethical approval was obtained from the Children’s Memorial Health Institute Bioethical Committee, Warsaw, Poland. The CONSORT flowchart is presented on Figure 2.

### Inclusion criteria

Patients had to fulfill all of the following inclusion criteria:

written consent of a patient (if at least 16 years old) and parents,

age: over 5 and below 19 years old,

overweight or obesity (BMI> 90pc according to IOTF BMI charts),

ALT activity ≥ 1,3 ULN (upper limit of normal),

hyperechogenicity of the liver on ultrasound or liver histology consistent with NAFLD/NASH (≥ 5% of hepatocytes with macrovesicular fat)

Hyper-ALT and liver steatosis on ultrasound are the most common diagnostic criteria for NAFLD used in the clinical practice. However usually the grade of ALT elevation is not defined. To increase sensitivity and specificity of diagnosis of NAFLD we used combined criteria of 1,3 ULN of ALT and liver steatosis on ultrasound. Similar diagnostic criteria were previously applied in pediatric trials on NAFLD [22,23].

### Exclusion criteria

To be eligible to participate in the trial patients had to undergo thorough screening of any other conditions that might mimic the diagnosis of NAFLD or could interfere with analysis of treatment efficacy. Exclusion criteria comprised of any pathologic conditions affecting liver as HBV, HCV infection, chronic and acute liver failure, cholestasis, metabolic diseases like α1-antitripsin deficiency, Wilson disease, diabetes mellitus, hypothyroidism, alcohol consumption etc.

Exclusion criteria:

General criteria:

•Age < 5 or > 19 years

•Current or history of significant alcohol consumption

•Unlikely to co-operate in the study, to comply with study treatment or with the study visits.

Medical and therapeutic criteria:

1. Concerning liver diseases:

•Past or active HBV infection

•Past or active HCV infection

•Chronic or acute liver failure

•Cholestasis

•Wilson disease

•Cystic fibrosis

•α1-antitripsin deficiency

•Autoimmune hepatitis

•Tyrosinemia

•Galactosemia

•Beta-oxidation disorders

•Any recognized liver disease presenting with elevated ALT other than NAFLD/NASH

2. Concerning other concomitant diseases or associated clinical conditions:

•History of diabetes mellitus

•Familiar hypercholesterolemia

•Hypothyroidism

•Current or history of craniopharyngioma

•Any medical condition that considered by physician can unable completion of the trial

3. Concerning concomitant medications:

•Treatment with vitamin E, statins, UDCA, probiotics or metformin within 3 months prior to randomization

•Pharmacological treatment of hypertension within 3 months prior to randomization

•History of parenteral nutrition

In accordance to the recent recommendations of the European Society of Pediatric Gastroenterology, Hepatology and Nutrition (ESPGHAN) on NAFLD diagnostics [24], liver biopsy was proposed to the patients in whom elevation of ALT lasted for over 6 months during weight reduction therapy.

### Treatment groups

Patients who were selected to participate in the study and gave their written consent were randomly assigned to one of the two therapeutic groups:

▪ Omega-3 group: 450-1300 mg omega-3 fatty acids (containing DHA and EPA in 3:2 proportion) in two doses per day, to be taken with morning and evening meal. The dose of omega-3 was dependent on patient’s weight (see Table 1).

**Table 1:** Dosage of omega-3 fatty acids

**Body weight [kg]**	**Approx. total omega-3 daily dose [mg]**	**DHA daily dose [mg]**	**EPA daily dose [mg]**	**Dosage scheme**
**Under 40**	450	267	177,5	Twice a day
**40-60**	900	534	355	Twice a day
**Over 60**	1300	800	532,5	Twice a day

▪ Placebo/comparator group: the same dose of sunflower oil (containing omega-6 LC-PUFA).

Planned duration of the treatment is 24 weeks. Omega-3 and placebo are administered orally twice a day in the same dose and formulation of brown-colored and oval-shaped capsules. Capsules used in this study contain omega-3 LC-PUFA which was originally obtained from the marine algae. Study drugs were manufactured and blinded by Hasco-Lek, Poland® from the composition of the following ingredients: Incromega DHA 500 TG SR, Croda, Poland® (consisting of 100 mg/g EPA and 500 mg/g DHA) and Incromega EPA 500 TG SR, Croda, Poland® (consisting of 500 mg/g EPA and 100 mg/g DHA).

Throughout the whole treatment period (6 months) and next 6 months of the follow-up patients are advised to lose weight based on modification of their lifestyle (low-calorie diet and physical exercise). Pharmacological agents e.g. vitamin E, metformin, thiazolidinediones, statins, UDCA, probiotics or any that may affect body weight reduction, insulin resistance, lipid profile or fatty liver are not allowed to use during the trial.

### Dietary assessment

Simultaneously with pharmacological treatment, each patient received individually tailored dietary recommendations aimed to reduce body weight.

Detailed information about current quantity and quality of the patient’s diet was gathered by an experienced dietician in every center. Dieticians analyzed intake of total energy, essential nutrients as proteins, carbohydrates (including sucrose), fiber, lipids, main minerals, lipid- and water-soluble vitamins, cholesterol, saturated fatty acids, monounsaturated fatty acids and LC-PUFA including α-linoleic acid (ALA), linolenic acid (LA), arachidonic acid (ARA), DHA and EPA.

Various assessment methods including food records and dietary recalls are subjected to considerable error and bias, and none of these is considered as a 'gold standard' [25]. In our study dietary data were collected from patients and their parents using 3-days record (3dr) and food frequency questionnaire (FFQ). To assess portion size we used Album of Photographs of Food Products and Dishes by National Food and Nutrition Institute in both methods.

#### 3-day food record

3-day food record is commonly used and well recognized method of dietary assessment. It requires reporting all the food and beverages consumed for three consecutive days (2-week days and 1-weekend day).

All participants of the study had to complete 3-day food record at week 0 (M0). They were previously instructed by a dietician how to fill in the food records. Patients were asked to specify the volume of consumed food with usual household measurements (e.g. cups, tablespoons) or to indicate the weight of commercial products with information recipes. After completing the food record, participants met with the dietician to review all the information for record accuracy, completeness and portion size of individual items. When needed, food models were used.

#### Food Frequency Questionnaire (FFQ)

FFQ inquires about food habits of certain time period and it is based on defined list of typical food items [26]. In comparison to other records FFQ comprises longer time of dietary intake, imposes less burden on patients and is quick and easy to complete. However the precision in quantifying intakes may be questioned. FFQ is preferred method in large cross-sectional and cohort studies in children.

For the purpose of our study FFQs were administered by registered dieticians who interviewed children and their parents in all centres. The questionnaire was modified in order to get key information about food ingredients vital for the development of obesity and metabolic syndrome e.g. saturated fatty acids, carbohydrates and antioxidants and on the other hand, these that protect organism from metabolic syndrome development, such us unsaturated fatty acids and antioxidants. Dieticians used food models for an approximate estimation of the portion consumed by the patient. The list contained over 300 products: typical dishes, raw materials, processed food, fluids and dietary supplements. Participants were asked about frequency of intake for different food during the last month in terms of day, week or month. Where available they had to provide recipes for consumed products. Parents were supporting the minors in completing the record. The FFQ was performed at the beginning (week 0) and the end of study medication treatment (week 24).

Data of consumed food were calculated to 1 day amount intake and recalculated into nutrients and energy by Energia FFQ software by Andrzej Miegoc®. It offered a database of nutritional value of food and products as well as option to add new food items and recipes.

#### Dietetic intervention

All patients were encouraged to apply to their individually defined diet, which in combination with increased physical activity, aimed at slow reduction of body weight (approximately 0,5 kg per week) [27]. As calculated, this can be achieved with daily energy deficit of about 500 kcal. The main criterion for efficacy of our dietary intervention was the body weight loss by at least 5%.

Based on the data from dietary records (3 day food record and FFQ) dieticians instructed patients about the necessary dietary modifications and habits they had to adhere.

Patients received the following dietary recommendations:

low-calorie diet

maximum 5% of total energy from simple carbohydrates

dietary glycemic index <50

30% of the total energy from lipids with proportion of saturated/monounsaturated/polyunsaturated fatty acids as 1:1:1

adequate protein intake

The advised caloricity of the diet varied from 1500 to 1900kcal per day with respect to patient’s age, gender and level of physical activity to achieve expected weight reduction of 0,5 kg per week. Participants received examples of menus that matched necessary energy intake. Patients were aware to measure their body weight every week. In case of excessive weight gain the dietician might suggest decreased calorie intake and/or increased physical activity.

### Physical activity assessment

To assess the level of physical activity and sedentary lifestyle we used the modified International Physical Activity Questionnaire (IPAQ). This document is a open access, publically available surveillance system that was validated on large cohorts in a number of studies [28]. Answers to a set of questions assign the participant to a certain physical activity level (low, moderate or high). The IPAQ protocol is designed to be self-reported by a patient, however in our study parents assisted children in its completion.

### Outcome measures

Primary outcome measure of the study is a number of patients in whom ALT level decreased by minimum 0,3 ULN (upper limit of normal) after the 6 months of treatment with omega-3 when compared to the placebo group.

Secondary outcomes include the following parameters compared between omega-3 and placebo group after 6 months of treatment:

▪ normalization of ALT activity at week 24

▪ decrease of ALT, AST, GGTP and other liver function tests,

▪ normalization or improvement of liver steatosis on ultrasound based on Saverymuttu scoring system [29],

▪ changes in the level of fasting glucose and insulin, cholesterol and lipid profile,

▪ changes in insulin resistance markers as insulin, HOMA-IR, adipokines,

▪ alterations in the basic hematology and biochemical lab tests,

▪ difference in fat and lean body mass measurements (using electric bioimpedance TANITA® Body Composition Analyzer BC 418MA),

▪ caloric intake as well as intake of fats, fatty acids and sucrose.

Other outcomes comprise of changes in anthropometric measures of body weight (e.g. height, hip/waist circumference, triceps, subscapular and abdominal skinfolds thickness) as well as vital signs like heart rate and blood pressure.

In selected subgroups of patients we will also examine effect of treatment on:

▪ serum antioxidants e.g. glutathione, glutathione peroxidase, tromboxane,

▪ cytokines e.g. TNF-α

▪ fatty acid composition of plasma phospholipids [%wt/wt],

▪ bone mass density and fat content using dual x-ray absorptiometry (DEXA) (Lunar Prodigy, General Electric Healthcare, United States) - in a subgroup of patients who were eligible for this investigation in 2 centers,

▪ liver imaging by MR and fat content on 1H-MRS - in subgroups of patients who were eligible for these investigations,

▪ adipose tissue distribution on MRI - in a subgroup of patients who were eligible for this investigation in 1 center.

### Sample size

We decided to use decrease of ALT by 0.3 upper limit of normal value as the primary endpoint of the study. It was estimated that a sample size of 80 patients would be sufficient to reveal a difference in the treatment effect of 30% (45% in experimental group vs. 15% in control group) considering α = 0.05 and a power (β) of 80%. The number of 80 children accounted for approximately 20% withdrawals or losses. The sample size was calculated with StatDirect software version 2.7.9 (StatsDirect Ltd., England, UK).

### Statistical analysis

Data will be presented as medians with quartile range. The Mann–Whitney U test will be used for comparisons of median values between groups. The calculations will be performed with PASW Statistics 18 version 18.0.0, Polar Engineering and Consulting®.

For the categorical data analysis (for the primary end point and selected secondary end points) data will be organized in contingency tables, tested for difference between observed and expected values and appropriate tests will be applied to test the null hypothesis of no difference between the groups (chi-square or Fischer’s exact test) with StatsDirect version 2.7.9 (StatsDirect Ltd., England, UK). The differences will be regarded significant at p<0.05. Statistical methods will be applied for intention-to-treat and per protocol analysis.

## Conduct of the trial

### Study visits overview

The study will be divided into the selection period, treatment period and the follow-up.

In the selection period patients were carefully screened to confirm diagnosis of NAFLD and to identify any potential exclusion criteria.

On the first visit at week 0 (M0) patients with NAFLD undergo wide-range physical, anthropometric, laboratory and imaging investigations. When eligible they are included in the study, randomized and receive study drug or placebo. At the same time patients are consulted by dietician, report 3-day food record, FFQ and IPAQ. They are instructed about their individual low-calorie diet and how to keep increased level of physical activity.

During the treatment period next two visits are planned:

M1 at 12 weeks after the inclusion,

M2 at 24 weeks after the inclusion.

At visit M1 a routine physical examination along with simplified laboratory tests and anthropometry are done. Patients report their complaints and adverse events to the physician and consult their diet with a dietician.

At visit M2 patients undergo physical examination, comprehensive laboratory and imaging examinations. Repeated food and physical activity questionnaires are recorded. Patients stop taking the study drug however the lifestyle recommendations are further maintained.

A follow-up visit (M3) is scheduled 6 months after the end of therapy to check whether changes of body weight, laboratory parameters of liver function and imaging are sustained.

Blood tests measurements perform the local laboratories, except for antioxidants, cytokines and fatty acids profile that are sent to the central laboratory. Upper limit of normal of ALT activity varies between the four medical centers (40–45 U/L), therefore in the statistical analysis we will calculate and use a percentage of the ULN.

Each anthropometric parameter and vital sign was measured twice and the mean value was taken into statistics. Systolic/diastolic blood pressure and heart rate were measured by the automatic blood pressure device (Dinamap XL, Critikon®, U.S.). Patients rested for 10 minutes in the supine position before measurement. Arm circumference was set midway between the shoulder tip and the olecranon process. The same arm was used throughout the study.

One experienced radiologist was performing abdominal ultrasound at each centre and estimated the degree of liver steatosis according to Saverymuttu scoring system [29]. When applicable, liver biopsy was done and assessed by two independent pathologists. At each centre a registered dietician administered food records and monitored adherence to the dietary regime.

At every visit any adverse events (AEs), that have occurred since the last visit and changes in the concomitant treatment were documented. For all investigations the data were entered into electronic case report form (eCRF) system and stored there anonymously.

The detailed schedule of the trial visits and investigations are summarized in Table 2.

**Table 2:** Investigation schedule

**Procedure**	**Inclusion**	**Treatment period**		**Follow-up**
	**M0**	**M1**	**M2**	**M3**
	**Week 0**	**Week 12**	**Week 24**	**Week 48**
**Written consent**	X			
**Medical history**	X	X	X	X
**Randomization**	X			
**Study drug dispensing**	X	X		
**End of study drug treatment**			X	
**Compliance**		X	X	X
**Adverse events monitoring**		X	X	X
**Previous and concomitant treatment**	X	X	X	X
**Vital signs and physical examination**	X	X	X	X
**Anthropometry + TANITA® bioimpedance**	X	X	X	X
**Abdominal utrasound**	X		X	X
^**a**^**Liver biopsy**	X			
**Dietary assessment:**				
- dietetic counseling	X	X	X	X
- 3 day food record	X			
- FFQ	X		X	
**Physical activity:**				
- general counselling	X	X	X	X
- IPAQ	X		X	
**Laboratory tests:**				
- hematology	X		X	
- ALT,AST, GGTP, bilirubin, INR	X	X	X	X
- fasting glucose, insulin	X	X	X	X
- OGTT glucose, insulin 0-240mins	X			
- fasting lipids profile	X		X	X
- adipokines, cytokines, antioxidants	X		X	
- fatty acid composition of plasma phospholipids	X		X	
^**a**^**DEXA**	X		X	
^**a**^**MRI - body fat distribution**	X		X	
^**a**^**1H-MRS - liver fat content**	X		X	

^a^Done in selected patients.

Vital signs: mean BP, HR and temperature.

Anthropometry includes measurements of body weight and height, waist, hips and mid upper arm circumference (IOTF, WHO), triceps, abdominal and subscapular skin fold thickness.

OGTT glucose and insulin measurements were performed in intervals every 30 minutes from 0 to 240 minutes.

### Randomization

Eligible participants who fulfilled inclusion criteria were randomized into the trial. Using computer statistical software StatsDirect version 2.7.9 (StatsDirect Ltd., England, UK) the list of random treatment assignments was generated. Randomization was made to two arms (omega-3 or placebo), in blocks of four individuals, stratified by centre. The use of centre stratification guarantees balance and increases statistical accuracy. Investigators sent randomizations requests by fax to the central randomization center (CRC) responsible for the process of randomization. In return they received a random number that was subsequently allocated to the choice of one of the products (numbered 100 or 101).

### Treatment dispensing and assessment of compliance

All the study treatments were provided in a capsule form. Identical appearance of capsules for both arms (omega-3 and placebo) ensured the double blind study design. Study drug were stored in plastic boxes containing 200 capsules at room temperature. The boxes were the same for the two treatments arms, blinded by a manufacturer, marked with 3 digits: “100” or “101” – for omega-3 or placebo.

Study treatment was dispensed by the investigator at the inclusion visit M0. After the validation of patient’s eligibility the investigator contacted with the CRC to declare the inclusion of the patient and allocate his/her therapeutic unit number. The kit number attributed by CRC corresponded to the randomized treatment arm. The box marked with respective kit number were given to the patient.

At the visit M1 the treatment pack allocated at the previous visit was retrieved from the patient and the drug accountability of the remaining capsules was performed.

At the final M2 study visit the kits allocated at visits M0 and M1 were retrieved from the patient. The number of used and remaining capsules were documented to estimate the compliance with the study drug. When the study had been completed no new allocation of treatment was made.

## Results

Patients recruitment for the trial started in 2008 and ended in 2011. From a total of 86 screened participants 10 were found ineligible (11.6%). The most frequent reason for ineligibility was ALT activity <1.3x ULN. A total of 76 children with NAFLD met the inclusion criteria and were randomly assigned to receive either the omega-3 (n=38) or placebo (n=38).

The follow-up of the study is still ongoing. We are currently finalizing data collection obtained after 24 weeks of treatment. The full statistical analysis of the study results will be completed in the mid-2013.

## Discussion

Marine omega-3 fatty acids are very promising dietary supplements used in prevention and therapy of cardiovascular, inflammatory, immunological, psychological and neurological disorders. They are safe pharmacological agents within the wide range of dose, what was shown in several studies including pregnancy, newborns and elderly and confirmed by the European Food Safety Authority (EFSA) in 2012 [30].

The beneficial effects of omega-3 fatty acids are possibly secondary to their anti-inflammatory, antithrombotic, antiarrhythmic, hypolipidemic and vasodilatory properties [16-19,31-38]. There is an evidence they could improve lipid prolife by lowering triglycerides, decrease insulin resistance and cytokine synthesis [19,34-38]. These effects may be linked with the pathogenesis of NAFLD. Already a number of studies showed efficacy of omega-3 fatty acids in the metabolic syndrome-related conditions [37-41]. Therefore omega-3 fatty acids may be potentially promising medication in the treatment of NAFLD.

To our best knowledge, our study is one of the very few double-blind, placebo-controlled randomized trials testing efficacy of omega-3 in children with NAFLD. Previously Nobili et al. supplemented a group of 60 children with either DHA or placebo for 6 months. The therapy decreased liver steatosis on ultrasound, improved triglycerides and insulin resistance markers, however no change in ALT activity was observed [21]. Further pediatric trials supporting or refuting use of omega-3 fatty in NAFLD are needed.

The review of the literature of randomized trials suggests that wide-range doses (0,8-13,7g per day) of omega-3 fatty acids are safe and may be efficient in NAFLD, however the optimal dose and proportion in the treatment of NAFLD has not been agreed yet [20]. Moreover the evidence is limited in children. We used omega-3 formula containing EPA and DHA, that should combine positive metabolic (mostly EPA) and anti-inflammatory effects (DHA). The composition of the studied supplement consisted of pre-defined DHA/EPA ratio (3:2). The chosen dose and content of omega-3 in our study was decided on the basis of review of the promising results from available open-label studies and it is a compromise that should provide safety and efficacy of therapy [42]. However, the study is testing only one dose of omega-3 instead of multiple doses to determine the dose–response relationship. Use of sunflower oil as a placebo/comparator may be justified because omega-6 are commonly present in a regular diet and the given dose does not influence their nutritional balance.

Weight-dependent dosing may be controversial, but it has to be regarded that the participants are children aged from 6 years to 19 years. The body mass might vary largely in this cohort, therefore differentiation between pediatric and adult doses can be explained. We subjectively set the weight margin at 40 kg and 60 kg for dosage of omega-3 but there are also several studies in children supplementing omega-3 for various indications in weight-dependent manner (per range and per kg) [43,44].

6-month duration of treatment is frequently applied in clinical trials with dietetic supplements like fish oil and it is considered sufficient to achieve therapeutic results and maintain satisfactory compliance [9,13,20].

The strength of our study is a well designed randomization and data collection system, clearly defined dietary counseling and a wide-range of parameters tested. In most previous trials investigating pharmacological treatment for NAFLD, dietetic surveillance was poorly described.

Several methods for assessment of body composition were used in our study (e.g. anthropometry, bioelectric impedance, MRI and DEXA). Among them DEXA becomes the gold standard method giving accurate and noninvasive measure of bone, fat distribution, and muscle tissues during single procedure of total body scan. Data obtained from all these techniques will be studied to estimate the efficacy of treatment and compared between each other to select the most reliable method reflecting changes of body composition in our patients.

The limitation of our study is the definition of NAFLD which is based on ultrasound and ALT (both criteria fulfilled together). Still, this allows to enroll those who present with significant steatosis (as indicated by ultrasound) and possibly inflammation (as indicated by ALT). Liver biopsy is rarely used to select children with NAFLD and for assessment of therapy as there are limited indications for liver biopsy in children.

To minimize possible bias we designed randomized, double-blind trial. Study treatment and placebo have identical appearance of capsules and packaging in plastic pill boxes to ensure the double blind study design. Data of the patients are entered and organized in the electronic case report form (e-CRF). The treatment allocation to the patients is done using a centralized randomization. Randomization is fixed, balanced and stratified according to centre. Both e-CRF and randomization are carried by the external company. Ultrasound was performed each time by the same radiologist, histology of the liver was assessed by two independent pathologists. Advanced lab tests are sent and examined in the central laboratory.

As there is no efficient pharmacological treatment for NAFLD, the positive result of this randomized trial will place omega-3 fatty acids as a useful agent in the treatment of NAFLD in children which can be safely applied together with lifestyle modification. No or negative effect of supplementation will add valuable evidence against its use in NAFLD. Nevertheless, final findings are expected to strongly contribute to the knowledge base and public health guidelines regarding use of omega-3 fatty acids in NAFLD, metabolic syndrome and obesity-related diseases.

## Competing interests

All authors declare that they have no competing interests.

## Authors’ contributions

WJ participated in the study design and coordination of the study, drafted and revised the manuscript. PS participated in the study design and coordination of the study, drafted and revised the manuscript. DL participated in the study design and revised the manuscript. AW participated in the design of laboratory examinations. AR participated in the study design. JNM participated in the design of dietetic intervention. PW participated in the study design. All authors read and approved the final manuscript.

## Authors’ information

PS is a Chairman of the Hepatology Committee of the European Society for Pediatric Gastroenterology and Nutrition (ESPGHAN) & President of the Polish Pediatric Society of Gastroenterology, Hepatology and Nutrition. WJ, DL are members of the Polish Pediatric Society of Gastroenterology, Hepatology and Nutrition.

## Pre-publication history

The pre-publication history for this paper can be accessed here:

http://www.biomedcentral.com/1471-2431/13/85/prepub
